# Assessing organ at risk position variation and its impact on delivered dose in kidney SABR

**DOI:** 10.1186/s13014-022-02041-2

**Published:** 2022-06-27

**Authors:** Mathieu Gaudreault, Shankar Siva, Tomas Kron, Nicholas Hardcastle

**Affiliations:** 1 Department of Physical Sciences, Peter MacCallum Cancer Centre, 305 Grattan St, Melbourne, VIC 3000 Australia; 2 Sir Peter MacCallum Department of Oncology, The University of Melbourne, Melbourne, VIC 3000 Australia; 3 Department of Radiation Oncology, Peter MacCallum Cancer Centre, Melbourne, VIC 3000 Australia; 4 Centre for Medical Radiation Physics, University of Wollongong, Wollongong, NSW 2522 Australia

**Keywords:** SABR, Kidney, Interfraction, Intrafraction, ART

## Abstract

**Background:**

Delivered organs at risk (OARs) dose may vary from planned dose due to interfraction and intrafraction motion during kidney SABR treatment. Cases of bowel stricture requiring surgery post SABR treatment were reported in our institution. This study aims to provide strategies to reduce dose deposited to OARs during SABR treatment and mitigate risk of gastrointestinal toxicity.

**Methods:**

Small bowel (SB), large bowel (LB) and stomach (STO) were delineated on the last cone beam CT (CBCT) acquired before any dose had been delivered (PRE CBCT) and on the first CBCT acquired after any dose had been delivered (MID CBCT). OAR interfraction and intrafraction motion were estimated from the shortest distance between OAR and the internal target volume (ITV). Adaptive radiation therapy (ART) was used if dose limits were exceeded by projecting the planned dose on the anatomy of the day.

**Results:**

In 36 patients, OARs were segmented on 76 PRE CBCTs and 30 MID CBCTs. Interfraction motion was larger than intrafraction motion in STO (p-value = 0.04) but was similar in SB (p-value = 0.8) and LB (p-value = 0.2). LB was inside the planned 100% isodose in all PRE CBCTs and MID CBCTs in the three patients that suffered from bowel stricture. SB D0.03cc was exceeded in 8 fractions (4 patients). LB D1.5cc was exceeded in 4 fractions (2 patients). Doses to OARs were lowered and limits were all met with ART on the anatomy of the day.

**Conclusions:**

Interfraction motion was responsible for OARs overdosage. Dose limits were respected by using ART with the anatomy of the day.

**Supplementary Information:**

The online version contains supplementary material available at 10.1186/s13014-022-02041-2.

## Introduction

Stereotactic ablative body radiotherapy (SABR) is a novel treatment to treat renal cell cancer (RCC) in medically inoperable patients, resulting in excellent local control and low toxicities [1, 2]. However, the large dose received to organs at risk (OARs) surrounding the target might result in undesirable toxicity post SABR treatment [3–6]. Patients will therefore benefit from any effort to minimise dose deposited to OARs.

Small bowel (SB), large bowel (LB) and stomach (STO) are OARs in the context of kidney SABR treatment. These organs are subject to daily positional and shape changes, which may result in variations between planned and delivered dose [7, 8]. Bowel and stomach motion include peristalsis motion and respiratory-induced motion, in addition to shape and size changes due to filling [9].

To account for motion, the use of an isotropic margin expansion to OAR contour to create a planning organ at risk volume (‘PRV’ technique) is recommended [8]. In the case of bowels, a structure container including the entire intestinal cavity (‘bowel bag’ technique) may be used [10, 11]. Moreover, adaptive radiation therapy (ART) may be a solution to account for OAR interfraction motion [12, 13].

Intrafraction motion and its impact on the dose distribution in kidney SABR have been studied in the literature [14–18]. In particular, kidney motion under free-breathing was reported to be less than 10 mm of amplitude [15]. However, intrafraction motion may lead to significant dose difference with respect to the static planned dose [17]. Abdominal OAR interfraction motion were reported in liver SBRT [19]. Dose limits to OAR were exceeded in stomach, heart, and oesophagus, mostly due to motion in the supero-inferior direction. However, the probability of small bowel and large bowel overdosage is expected to be higher in kidney SABR treatment compared with liver SBRT treatment.

We observed three cases of bowel stricture requiring surgery post kidney SABR treatment at our institution. The intent of this study is to investigate dosimetric factors that could have led to this toxicity. To do so, we aim to estimate intrafraction and interfraction variations of small bowel, large bowel and stomach and their impact on the delivered dose as determined from cone-beam CTs (CBCT) in treatment position. We further aim to suggest solutions that may lower dose received to OARs in kidney SABR treatment.

## Materials and methods

Toxicities of the whole kidney SABR cohort treated at our institution were not available at the time of this study. Rather, we selected for inclusion a contemporaneous cohort of kidney SABR patients to those we observed with bowel stricture. This was a pragmatic sample size based on availability of imaging data due to software upgrades. This included all 36 consecutive patients treated from January 2018 to February 2021 for whom online image guidance registration dicom files were available. Lesion sizes smaller or equal to 4 cm were prescribed 26 Gy in a single fraction (SF) and lesion sizes greater than 4 cm were prescribed 42 Gy in three fractions (MF) [20]. Renal metastasis were prescribed 20 Gy in a SF.

Each patient was simulated with a four-dimensional CT scan (4DCT) in free breathing on a Brilliance Big Bore 16-slice CT scanner (Philips Healthcare*,* Andover*, *MA*,* USA). Images were sorted into 10 phase-based bins with a bellows system for the respiratory trace (Philips Healthcare). The pixel spacing was either 1.17 mm or 1.37 mm. The slice thickness was 2 mm with the exception of two patients where 1 mm was used. The planning CT was the average intensity projection (AIP) of the 10 phase images in 30/36 patients, the AIP of the exhale phase images (typically phase 40% to phase 70%) in 5/36 patients who were treated with respiratory gating and a 3D exhale breath hold acquisition in 1/36 patient. Patients were immobilized at simulation and during treatment with the BodyFix vacuum drape (Elekta, Stockholm, Sweden).

The tumour was delineated on the planning CT to generate an internal target volume (ITV). The ITV covered the residual motion in the gating window as estimated on the 4DCT in respiratory gating cases and the estimated variation between repeat breath holds in the breath hold case. A planning target volume (PTV) was generated through a 5 mm isotropic expansion of the ITV. Dose distribution was optimized and calculated to the PTV by using the Eclipse treatment planning system (Varian Medical Systems, Palo Alto) with Photon Optimization algorithm (v15.6 or v15.1) for optimization and AcurosXB algorithm (v15.6 or v15.1) reporting dose to medium for dose calculation. The dose calculation grid was 2.5 mm or 1.25 mm in plane, and 2 mm in the supero-inferior direction. All patients were planned with volumetric modulated arc therapy (VMAT) with the exception of one patient planned with 3D conformal radiation therapy (3DCRT) and one patient with intensity-modulated radiation therapy (IMRT). According to our protocol, all plans underwent patient-specific QA pre-treatment that generally included a 4DCT review, treatment plan review and 3D calculation and delivered log file based pre-treatment QA with a 2%/2 mm Gamma passing rate.

Optimization was performed using bowel loops, bowel PRV, or the bowel bag contours. Clinical normal tissue dose constraints used are shown in the Additional file 1. Dose limits considered were based on the QUANTEC recommendations (SB D30cc < 12.5 Gy, LB D1.5cc < 26 Gy and STO D5cc < 22.5 Gy in the SF patient cohort and SB D0.03cc < 30 Gy, LB D1.5cc < 42 Gy, STO D0.03cc < 30 Gy, and STO D5cc < 22.5 Gy in the MF patient cohort) [21–23]. The metric LB D0.03cc was also investigated in both cohorts.

Interfraction motion may be due to daily variation in organ shape or size, weight loss during the course of treatment, radiation damage, or change in tumour size [24]. Interfraction positional change was measured from the CBCT acquired for setup at time of treatment; the CBCT acquired immediately prior to treatment was used (PRE CBCT). Intrafractional variation may be caused by respiratory motion, peristalsis, or cardiac motion [24]. Intrafraction motion was measured by using the first CBCT acquired after some dose had been delivered to the patient (MID CBCT). All CBCTs were acquired with either 125 kVp or 140 kVp with 2 mm slice thickness with the exception of 2 CBCTs with 1 mm slice thickness. The pixel spacing was either 0.91 mm or 0.51 mm.

SB, LB, and STO were retrospectively delineated in treatment position on all PRE CBCTs and MID CBCTs. Bowels were segmented by contouring each bowel loop independently from each other (‘bowel loop technique’). In the case of streak artefacts due to bowel gas motion during CBCT acquisition, organ edges were approximated. CBCT quality was classified qualitatively as ‘excellent’, ‘good’, and ‘approximate’ depending on how well bowel edges could be determined visually. The registration used to match the tumour on the CBCT to the planning CT performed by the radiation oncologist at time of treatment was applied, and the OAR contours on the CBCT were copied to the planning CT. Dose metrics were then extracted for each OAR based on their position on the planning CT, PRE CBCT, and MID CBCT.

Location of OARs was quantified through the determination of the shortest distance between the ITV and the OAR, denoted dist(ITV,OAR). In order to do so, the ITV contour was successively expanded with 1 mm isotropic margin. The overlap between the expanded ITV and OAR was determined after each expansion. The shortest distance between the two structures was defined as being the first distance in which the overlap between the two structures returned a non-null structure.

Interfraction motion was quantified by calculating the difference between the shortest distance on PRE CBCT and on planning CT, ${\Delta {\text{dist}}}_{{\text{CT}}}^{{\text{PRE}}}={\text{dist}}^{{\text{PRE}}}\left(\text{ITV},\text{OAR}\right)-{\text{dist}}^{{\text{CT}}}(\text{ITV},\text{OAR})$. An OAR closer to the ITV on PRE CBCT compared with its distance on CT had ${\Delta \text{dist}}_{{\text{CT}}}^{{\text{PRE}}}<0$. A similar quantity was defined to quantify intrafraction motion ${\Delta \text{dist}}_{{\text{PRE}}}^{{\text{MID}}}={\text{dist}}^{{\text{MID}}}\left(\text{ITV},\text{OAR}\right)-{\text{dist}}^{{\text{PRE}}}(\text{ITV},\text{OAR})$. The mean and standard deviation of the magnitude of the interfractional and intrafractional variation, $\left|\left.{\Delta \text{dist}}_{{\text{CT}}}^{{\text{PRE}}}\right|\right.$ and $\left|\left.{\Delta \text{dist}}_{{\text{PRE}}}^{{\text{MID}}}\right|\right.$, were reported.

To test if a variation in ${\Delta \text{dist}}_{{\text{CT}}}^{{\text{PRE}}}$ or ${\Delta \text{dist}}_{{\text{PRE}}}^{{\text{MID}}}$ leads to a variation in the planned dose per fraction to OAR, the Pearson correlation coefficient (r) between $\Delta \text{dist}$ and the difference between the near to maximum planned dose per fraction of this OAR on PRE CBCT and on planning CT, ${\Delta \text{D}0.03\text{cc}}_{{\text{CT}}}^{{\text{PRE}}}={\text{D}0.03\text{cc}}^{{\text{PRE}}}-{\text{D}0.03\text{cc}}^{{\text{CT}}}$, or on MID CBCT and on PRE CBCT, ${\Delta \text{D}0.03\text{cc}}_{{\text{PRE}}}^{{\text{MID}}}={\text{D}0.03\text{cc}}^{{\text{MID}}}-{\text{D}0.03\text{cc}}^{{\text{PRE}}}$, was calculated.

In the case where a dose limit was exceeded in a given fraction by using structures on PRE CBCT, a new treatment plan was generated to investigate if dose to organs could have been lowered while preserving adequate target coverage. To do so, dose optimization and calculation was first performed by using contours determined on the PRE CBCT to perform the optimization with the objectives used in the original treatment plan. If constraints were still not met, a knowledge-based planning (KBP) model (RapidPlan v15, Varian Medical Systems, Palo Alto) was used to further improve the model (KBP model was not available at time of original treatment planning). Metric extraction, determination of the shortest distance between the ITV and OAR and dose optimization and calculation were performed by using the Eclipse Scripting Application Programming Interface (ESAPI v16.1, Varian Medical System, Palo Alto).

Statistical significance of the difference in the medians was determined with a Wilcoxon signed rank test for equal sample size and a Wilcoxon rank sum test otherwise by using the Scipy v1.5.2 module. The null hypothesis was rejected at the 5% significance level (p-value < 0.05).

## Results

**Fig. 1:**
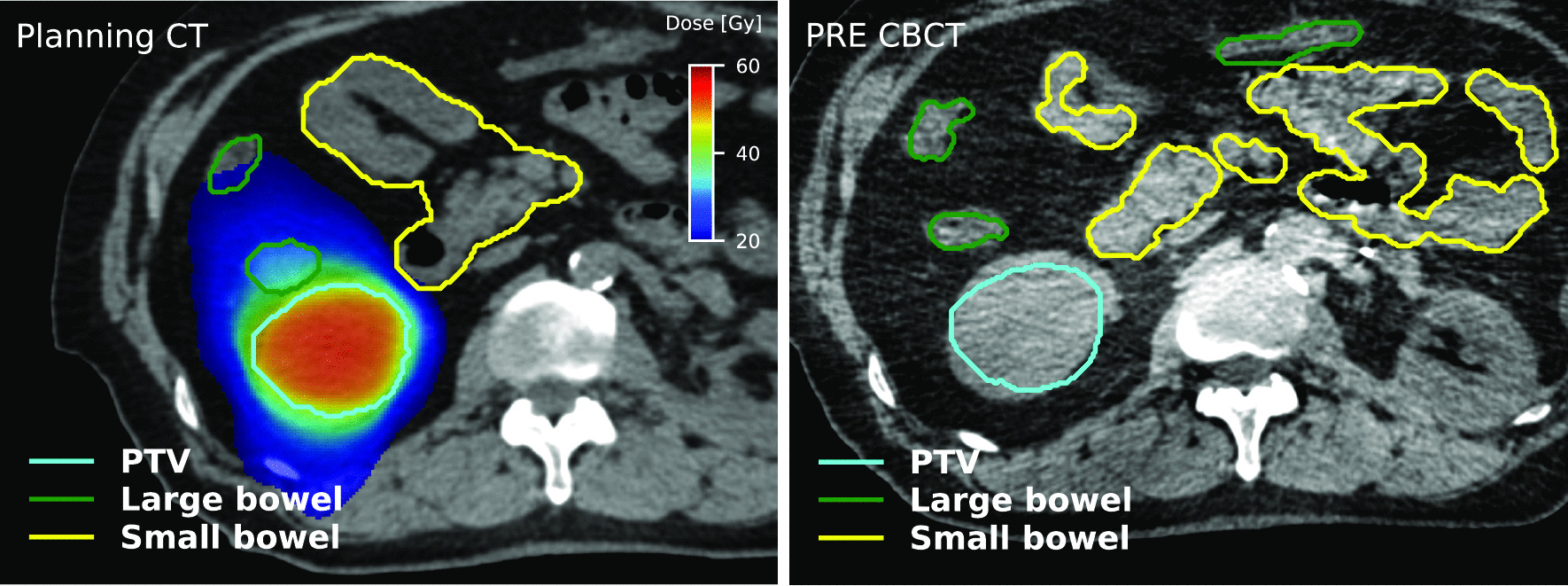
Illustration of the dose distribution and location of the PTV (cyan), LB (green), and SB (yellow) on the planning CT (left) and PRE CBCT (right) for one patient

The characteristics of the 36 patients that received kidney SABR considered in this study are described in Table 1. In this cohort, 34 lesions were primary renal cell cancer, 1 lesion was a renal metastasis, and 1 lesion was a renal bed. A total of 76 PRE CBCTs were acquired. MID CBCTs were acquired in 12 patients in the SF cohort and in 9 patients (18 Fx) in the MF cohort, for a total of 30 MID CBCTs. The time difference between MID CBCT and PRE CBCT ranged from 5.5 min to 22.8 min (mean ± standard deviation = 9.6 ± 3.8 min). Optimisation was performed to bowel loops in 20 plans (56%), PRV in 13 plans (36%) and bowel bag in 3 plans (8%). The quality was judged ‘excellent’/‘good’/‘approximate’ in 25%/41%/34% of all PRE CBCTs and 7%/23%/70% of all MID CBCTs. An illustration of the dose distribution and OARs location on the planning CT and PRE CBCT is shown in Fig. 1 (Patient 25).

**Table 1:** Patient’s characteristics and patient numbers included in this study

Patient characteristic	Description	n (patients)
Age at treatment	Median	74 y
	Range	52–87 y
Sex	Male	25
	Female	11
Fractionation	1 Fraction	16
	3 Fractions	20
Delivery technique	VMAT	34
	IMRT	1
	3DCRT	1
ECOG	Not available	7
	0	12
	1	15
	2	2
Treatment intent	Radical	34
	Palliative	2

### Positional variation

**Fig. 2:**
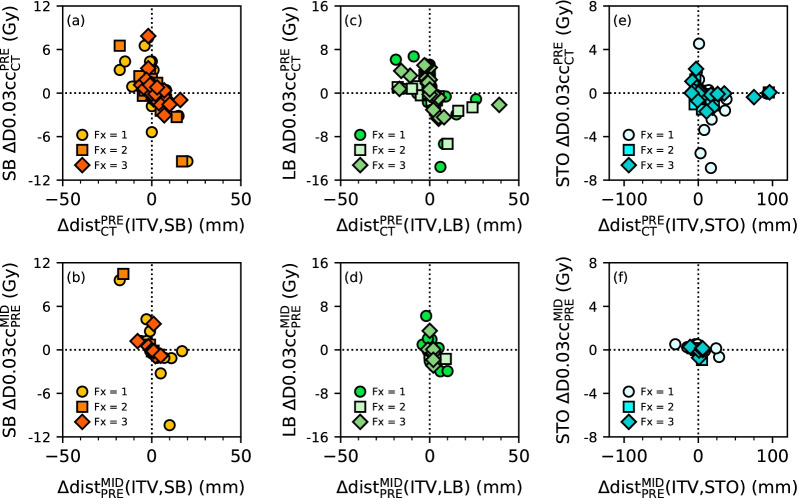
Estimated OAR interfraction motion (**a**), (**c**), (**e**) and intafraction motion (**b**), (**d**), and (**f**). OARs were closer to the ITV on CBCT if the shortest distance difference was negative and were closer to the ITV on the CT if the shortest distance difference was positive

Estimated interfraction motion and intrafraction motion are detailed in Table 2. All fractions considered, interfraction motion was larger in STO (n = 69) compared with SB (p-value < 10^−2^, n = 75) and LB (p-value < 10^−2^, n = 76). Difference in interfraction motion between SB and LB was not statistically significant (p-value = 0.2). Differences in interfraction motion between each fraction were not statistically significant for all three OARs (p-value = [0.06, 0.83]). The magnitude of the interfractional variation was $\left|\left.{\Delta \text{dist}}_{{\text{CT}}}^{{\text{PRE}}}\right|\right.$ = 4.4 ± 5.0 mm/5.1 ± 7.1 mm/12.9 ± 21.3 mm in SB/LB/STO. The near to maximum planned dose difference versus estimated interfraction motion of SB, LB, and STO are shown in Fig. 2a, c, e. Interfractional variation was anticorrelated with the near to maximum planned dose difference in SB (r = −0.7, p-value < 10^−9^), and in LB (r = −0.5, p-value < 10^−4^), but not in STO (p-value = 0.4).

**Table 2:** Statistical quantities of the estimated interfraction motion, ${\Delta \text{dist}}_{{\text{CT}}}^{{\text{PRE}}}(\text{ITV},\text{OAR})$, and estimated intrafraction motion, ${\Delta \text{dist}}_{{\text{PRE}}}^{{\text{MID}}}(\text{ITV},\text{OAR})$

OAR	Fx	n	Maximum (mm)	Q3 (mm)	Q2 (mm)	Q1 (mm)	Minimum (mm)
Estimated interfraction motion:${\Delta \text{dist}}_{{\text{CT}}}^{{\text{PRE}}}(\text{ITV},\text{OAR})$							
SB	All	75	20	4	0	− 2	− 18
	1	36	20	4	0	− 2	− 18
	2	20	17	3	0	− 2	− 18
	3	19	16	5.5	1	− 1.5	− 6
LB	All	76	39	2.25	0	− 2	− 19
	1	36	26	1	0	− 2	− 19
	2	20	24	3.25	0	− 3	− 18
	3	20	39	4.25	0	− 2	− 17
Stomach	All	69	96	14	3	− 1	− 9
	1	33	93	14	3	− 1	− 8
	2	18	95	8.25	3	− 0.75	− 5
	3	18	96	18.5	1	− 2	− 9
Estimated intrafraction motion:${\Delta \text{dist}}_{{\text{PRE}}}^{{\text{MID}}}(\text{ITV},\text{OAR})$							
SB	All	30	17	3	0	− 1	− 18
	1	18	17	4.5	0	− 1	− 18
	2	5	4	1	0	− 1	− 16
	3	7	5	1.5	1	− 1	− 8
LB	All	30	10	2	0	− 0.75	− 4
	1	18	10	3.75	0	− 1	− 4
	2	5	9	1	0	0	0
	3	7	2	2	2	0.5	− 1
Stomach	All	29	28	5	1	− 4	− 31
	1	18	28	4.75	− 0.5	− 4	− 31
	2	5	5	1	1	− 3	− 8
	3	6	6	5.75	3	1	− 11

The maximum, first, second and third quartile, and the minimum of the distributions are shown. Results are in mm

All fractions considered, intrafraction motion was smaller than interfraction motion in STO (p-value = 0.04) while differences between intrafraction and interfraction motions were not statistically significant in SB (p-value = 0.8) and in LB (p-value = 0.2). Intrafraction motion differences between each fraction were not statistically significant for the three organs (p-value between 0.1 and 0.7). The magnitude of the intrafractional variation was $\left|\left.{\Delta \text{dist}}_{{\text{PRE}}}^{{\text{MID}}}\right|\right.$ = 4.4 ± 5.2 mm/2.3 ± 2.6 mm/7.1 ± 7.9 mm in SB/LB/STO. The near to maximum planned dose difference of SB, LB, and STO versus estimated intrafraction motion are shown Fig. 2b, d, f. Intrafractional variation was anticorrelated with the near to maximum planned dose difference in SB (r = −0.8, p-value < 10^−5^, n = 30), LB (r = –0.6, p-value < 10^−3^, n = 30), and STO (r = −0.5, p-value < 10^−2^, n = 29).

### Adaptive therapy

**Fig. 3:**
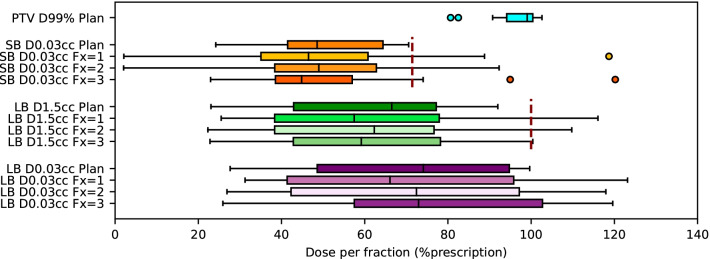
Boxplots of planned dose metrics PTV D99%, SB D0.03cc, LB D1.5cc and LB D0.03cc at fraction 1/2/3 for the multifraction patient cohort. Red dashed lines indicate dose limit

Patients 22, 35, and 36, all in the MF patient cohort, suffered from bowel stricture. Planned and estimated delivered dose volume histograms of all patients are available in the Additional file 2. In the context of adaptive therapy, only estimated doses on PRE CBCT were considered. All dose limits to OAR were respected in the SF patient cohort. Dose limits were exceeded in 5 patients in the MF patient cohort. Dose metrics PTV D99%, SB D0.03cc, LB D1.5cc, and LB D0.03cc of the MF cohort are shown in Fig. 3. Dose metric SB D0.03cc was not respected in 8 Fx (4 patients) and LB D1.5cc was not respected in 4 Fx (2 patients). The metric LB D0.03cc was larger than 100% of the prescription dose in 13 Fx (6 patients).

**Fig. 4:**
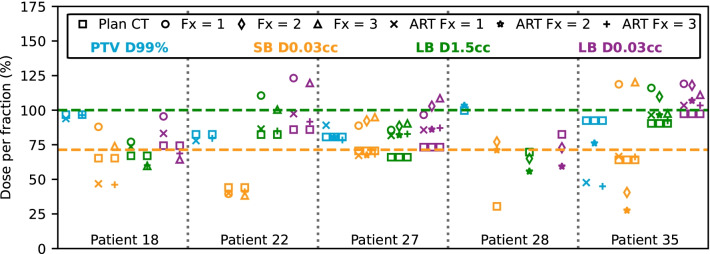
Planned dose metrics per fraction PTV D99%, SB D0.03cc, LB D1.5cc and LB D0.03cc at fraction 1/2/3 for patients where a dose limit was exceeded. Results from the planned dose projected on PRE CBCTs and from ART re-optimization are shown. Orange and green dashed line indicate dose limit to the small and large bowel, respectively

Dosimetric conflicts could have been solved with ART in all cases, as shown in Fig. 4. In Patient 35, the KBP model was used in the three fractions due to challenging small bowel and large bowel locations. Target coverage was compromised with ART (ART PTV D99% = 20.0 Gy/32.0 Gy/18.9 Gy compared with planned PTV D99% = 38.8 Gy) but doses to small bowel and large bowel were reduced (ART SB D0.03cc = 27.9 Gy/11.6 Gy/27.8 Gy compared with PRE CBCT SB D0.03cc = 49.9 Gy/17.0 Gy/50.5 Gy, and ART LB D1.5cc = 40.5 Gy/40.5 Gy/38.8 Gy compared with PRE CBCT LB D1.5cc = 48.7 Gy/46.1 Gy/41.0 Gy in Fx = 1/2/3).

In the remaining four patients (Patient 18, 22, 27, and 28), target coverage was reasonable with optimisation using the original objectives on the PRE CBCT contours (ART PTV D99% ranged from − 5% to 10% relative to planned PTV D99% in 7 Fx), and doses to OAR were all reduced (ART SB D0.03cc ranged from − 47% to − 8% relative to PRE CBCT SB D0.03cc in 6 Fx and ART LB D1.5cc = −22% and − 15% relative to PRE CBCT LB D1.5cc in 2 Fx).

## Discussion

Dose received by SB, LB and STO during kidney SABR treatment was estimated from the planned dose to the projected structures delineated on CBCT acquired before and during treatment. This study was motivated by reported cases of bowel stricture post SABR treatment at our institution. It is important to note that these three cases of the 36 cases analysed in this study do not represent a crude rate of this toxicity. Rather, we selected this cohort to estimate delivered bowel dose in a series of contemporaneously treated patients to those with bowel stricture.

Interfraction and intrafraction motion of SB, LB and STO were estimated from the shortest distance from these OARs to the ITV. Interfraction motion was larger than intrafraction motion in STO, while both motions were similar in SB and LB. A similar conclusion was achieved in an earlier investigation of abdominal OAR motion using MRI, except that interfraction motion was also larger than intrafraction motion in LB [25].

A correlation was observed between the near to maximum planned dose and the interfraction and intrafraction motion; as the OAR to ITV distance decreased, the near to maximum dose to the OAR increased. This correlation was not observed in a previous study in liver SBRT [19], which used the dose metric OAR D0.3cc and the Euclidian distance between centres of mass. The difference may be explained by the use of the shortest distance between the tumour and the OAR in this current study rather than the distance between their centres of mass, as the later can be influenced by OAR motion far from the ITV.

All dose limits were respected in the SF patient cohort. However, dose limit SB D0.03cc was exceeded in 4 patients (8 Fx) and LB D1.5cc was exceeded in 2 patients (4 Fx) in the MF cohort. These results are consistent with earlier findings where interfraction motion was responsible for abdominal OARs overdosage [19].

**Fig. 5:**
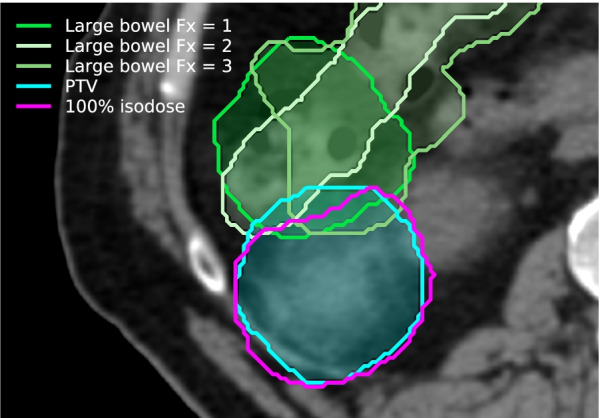
Large bowel (green) position as determined from PRE CBCT in fraction 1/2/3 of a patient that had bowel stricture surgery post SABR treatment. The PTV (cyan) and the 100% isodose line (magenta) are shown

LB was inside the planned 100% isodose in all PRE CBCTs and MID CBCTs acquired in patients that suffered from bowel stricture (Patient 22, 35, and 36). An example is shown in Fig. 5. In these patients, treatment planning was challenging as LB overlapped with the target. A PRV was used on the LB in all three patients, and the target coverage was compromised to meet the LB constraint applied to the PRV. Interfraction LB motion however resulted in portions of the LB moving into the 100% isodose line at time of treatment, indicating that the PRV margin was not sufficient to space the LB.

ART would have successfully reduced dose to OARs by using the anatomy of the day as optimization structures. However, online ART may increase significantly the total treatment time due to the additional planning time. Another solution would be to not deliver dose at a given fraction and postpone treatment if OAR locations differ from the original plan.

The main limitation in this study comes from CBCT quality. Reduced quality was mainly due to the presence of bowel gas generating streak artefacts, which can obscure the edge of organs. As a result, some contours were only an approximation of the actual location of OARs. Improvement of CBCT quality to reduce bowel gas artefact may be achieved with machine learning [26]. Furthermore, this problem may be avoided by magnetic resonance guided radiation therapy (MRgRT) [27, 28]. Moreover, due to the inferior CBCT quality and Hounsfield unit inaccuracy, dose calculation was not performed on the CBCT, but the dose as calculated on the planning CT was assumed for all CBCT dose assessments. This approximation may further be improved through use of improved on-treatment CBCT with reduced artefacts and improved HU accuracy [29, 30].

A further limitation is the unequal sampling frequency of interfraction (76 potential measurements) and intrafraction motion (30 potential measurements). The small number involved in the determination of the intrafraction motion may have affected the statistical significance of the results.

Finally, the intrafractional variation was measured by using only two points in time. This method can therefore capture positional drift but cannot provide a complete picture of respiratory motion or peristalsis, as higher temporal sampling would be needed.

## Conclusions

Interfraction motion was responsible for the overdosage of the large bowel in three kidney SABR patients that experienced bowel stricture post SABR treatment. Dose limits could have been respected by using adaptive radiation therapy with the anatomy of the day.

## Supplementary Information

**Additional file 1. Table S1**: Normal tissues dose constraints used.

**Additional file 2.** Dose Volume Histograms for all patients.

## Data Availability

The datasets used and/or analysed during the current study are available from the corresponding author on reasonable request.

## Abbreviations

3DCRT: 3D conformal radiation therapy
4DCT: Four-dimensional CT
AIP: Average intensity projection
ART: Adaptive radiation therapy
CBCT: Cone beam CT
Fx: Fraction
IMRT: Intensity-modulated radiation therapy
ITV: Internal target volume
LB: Large bowel
KBP: Knowledge-based planning
MF: Multifraction
MID CBCT: Cone beam CT during treatment
OAR: Organ at risk
PRE CBCT: Cone beam CT pre-treatment
PRV: Planning organ at risk volume
PTV: Planning target volume
SABR: Stereotactic ablative body radiotherapy
SB: Small bowel
SF: Single fraction
STO: Stomach
VMAT: Volumetric modulated arc therapy

## References

[1] Siva S, Pham D, Gill S, Corcoran NM, Foroudi F (2012). A systematic review of stereotactic radiotherapy ablation for primary renal cell carcinoma. BJU Int.

[2] Siva S, Louie A, Warner A, Muacevic A, Gandhidasan S, Ponsky L (2018). Pooled analysis of stereotactic ablative radiotherapy for primary renal cell carcinoma: A report from the International Radiosurgery Oncology Consortium for Kidney (IROCK). Cancer.

[3] Morias S, Marcu LG, Short M, Giles E, Potter A, Shepherd J (2018). Treatment-related adverse effects in lung cancer patients after stereotactic ablative radiation therapy. J Oncol.

[4] Goldsmith C, Plowman PN, Green MM, Dale RG, Price PM (2018). Stereotactic ablative radiotherapy (SABR) as primary, adjuvant, consolidation and re-treatment option in pancreatic cancer: Scope for dose escalation and lessons for toxicity. Radiat Oncol.

[5] Pollom EL, Deng L, Pai RK, Brown JM, Giaccia A, Loo BW (2015). Gastrointestinal toxicities with combined antiangiogenic and stereotactic body radiation therapy. Int J Radiat Oncol Biol Phys.

[6] Singh AK, Tierney RM, Low DA, Parikh PJ, Myerson RJ, Deasy JO (2006). A prospective study of differences in duodenum compared to remaining small bowel motion between radiation treatments: Implications for radiation dose escalation in carcinoma of the pancreas. Radiat Oncol.

[7] Kvinnsland Y, Muren LP (2005). The impact of organ motion on intestine doses and complication probabilities in radiotherapy of bladder cancer. Radiother Oncol.

[8] Muren LP, Smaaland R, Dahl O (2003). Organ motion, set-up variation and treatment margins in radical radiotherapy of urinary bladder cancer. Radiother Oncol.

[9] Mostafaei F, Tai A, Omari E, Song Y, Christian J, Paulson E (2018). Variations of MRI-assessed peristaltic motions during radiation therapy. PLoS ONE.

[10] Jabbour SK, Hashem SA, Bosch W, Kim TK, Finkelstein SE, Anderson BM (2014). Upper abdominal normal organ contouring guidelines and atlas: a Radiation Therapy Oncology Group consensus. Pract Radiat Oncol.

[11] Gay HA, Barthold HJ, O’Meara E, Bosch WR, el Naqa I, Al-Lozi R (2012). Pelvic normal tissue contouring guidelines for radiation therapy: a radiation therapy oncology group consensus panel Atlas. Int J Radiat Oncol Biol Phys.

[12] Glide-Hurst CK, Lee P, Yock AD, Olsen JR, Cao M, Siddiqui F (2021). Adaptive radiation therapy (ART) strategies and technical considerations: a state of the ART Review From NRG Oncology. Int J Radiat Oncol Biol Phys.

[13] Bertholet J, Anastasi G, Noble D, Bel A, van Leeuwen R, Roggen T (2020). Patterns of practice for adaptive and real-time radiation therapy (POP-ART RT) part II: offline and online plan adaption for interfractional changes. Radiother Oncol.

[14] Siva S, Pham D, Gill S, Bressel M, Dang K, Devereux T (2013). An analysis of respiratory induced kidney motion on four-dimensional computed tomography and its implications for stereotactic kidney radiotherapy. Radiat Oncol.

[15] Pham D, Kron T, Foroudi F, Schneider M, Siva S (2014). A review of kidney motion under free, deep and forced-shallow breathing conditions: implications for stereotactic ablative body radiotherapy treatment. Technol Cancer Res Treat.

[16] Al-Ward S, Wronski M, Ahmad SB, Myrehaug S, Chu W, Sahgal A (2018). The radiobiological impact of motion tracking of liver, pancreas and kidney SBRT tumors in a MR-linac. Phys Med Biol.

[17] Stemkens B, Glitzner M, Kontaxis C, de Senneville BD, Prins FM, Crijns SPM (2017). Effect of intra-fraction motion on the accumulated dose for free-breathing MR-guided stereotactic body radiation therapy of renal-cell carcinoma. Phys Med Biol.

[18] Prins FM, Stemkens B, Kerkmeijer LGW, Barendrecht MM, de Boer HJ, Vonken EJPA (2019). Intrafraction motion management of renal cell carcinoma with magnetic resonance imaging-guided stereotactic body radiation therapy. Pract Radiat Oncol.

[19] Schmid RK, Tai A, Klawikowski S, Straza M, Ramahi K, Li XA (2019). The dosimetric impact of interfractional organ-at-risk movement during liver stereotactic body radiation therapy. Pract Radiat Oncol.

[20] Siva S, Chesson B, Bressel M, Pryor D, Higgs B, Reynolds HM (2018). TROG 15.03 phase II clinical trial of Focal Ablative STereotactic Radiosurgery for Cancers of the Kidney—FASTRACK II 11 Medical and Health Sciences 1103 Clinical Sciences 11 Medical and Health Sciences 1112 Oncology and Carcinogenesis. BMC Cancer.

[21] Pan CC, Kavanagh BD, Dawson LA, Li XA, Das SK, Miften M (2010). Radiation-associated liver injury. Int J Radiat Oncol Biol Phys.

[22] Dawson LA, Kavanagh BD, Paulino AC, Das SK, Miften M, Li XA (2010). Radiation-associated kidney injury. Int J Radiat Oncol Biol Phys.

[23] Kavanagh BD, Pan CC, Dawson LA, Das SK, Li XA, ten Haken RK (2010). Radiation dose-volume effects in the stomach and small bowel. Int J Radiat Oncol Biol Phys.

[24] Wysocka B, Kassam Z, Lockwood G, Brierley J, Dawson LA, Buckley CA (2010). Interfraction and respiratory organ motion during conformal radiotherapy in gastric cancer. Int J Radiat Oncol Biol Phys.

[25] Henke LE, Green OL, Curcuru A, Lu Y, Mutic S, Parikh PJ (2018). Magnitude and dosimetric impact of inter- and intra-fraction anatomic variability during MRI-Guided Online Adaptive Radiation Therapy (MRgART) to the abdomen. Int J Radiat Oncol Biol Phys.

[26] Harms J, Lei Y, Wang T, Zhang R, Zhou J, Tang X (2019). Paired cycle-GAN-based image correction for quantitative cone-beam computed tomography. Med Phys.

[27] Keller B, Bruynzeel AME, Tang C, Swaminath A, Kerkmeijer L, Chu W (2021). Adaptive magnetic resonance-guided stereotactic body radiotherapy: the next step in the treatment of renal cell carcinoma. Front Oncol.

[28] Tetar SU, Bohoudi O, Senan S, Palacios MA, Oei SS, van der Wel AM (2020). The role of daily adaptive stereotactic mr-guided radiotherapy for renal cell cancer. Cancers.

[29] Zhao W, Vernekohl D, Zhu J, Wang L, Xing L (2016). A model-based scatter artifacts correction for cone beam CT. Med Phys.

[30] Chen L, Liang X, Shen C, Jiang S, Wang J (2020). Synthetic CT generation from CBCT images via deep learning. Med Phys.

