# Supplementary material for: Assessing organ at risk position variation and its impact on delivered dose in kidney SABR
Source: Radiat Oncol. 2022 Jun 27;17:112. doi: 10.1186/s13014-022-02041-2 (PMC9235197; doi:10.1186/s13014-022-02041-2)
Supplement: Supplementary file 1 — Additional file 1. Table S1: Normal tissues dose constraints used. [file 13014_2022_2041_MOESM1_ESM.docx]

|  | **26Gy/1Fx** | **42Gy/3Fx** |
| --- | --- | --- |
| Spinal coord | D0.03cc *<* 12 Gy | D0.03cc *<* 18 Gy |
| Skin | D1.5cc < 18 Gy | D1.5cc *<* 24 Gy |
| Small bowel | D30cc *<* 12.5 Gy | D0.03cc *<* 30 Gy |
| Large bowel | D1.5cc < 26 Gy | D1.5cc *<* 42 Gy |
| Stomach |  | D0.03cc *<* 30 Gy |
|  | D5cc < 22.5 Gy | D5cc < 22.5 Gy |
| Liver | No constraint | D700cc < 15 Gy |
| Contralateral kidney | V10Gy *<* 33% | V10Gy *<* 33% |

SUPPLEMENTAL MATERIAL

Table 1. Normal tissue dose constraints used.
